# The effect of low shear force on the virulence potential of *Yersinia pestis*: new aspects that space-like growth conditions and the final frontier can teach us about a formidable pathogen

**DOI:** 10.3389/fcimb.2012.00107

**Published:** 2012-08-09

**Authors:** Jason A. Rosenzweig, Ashok K. Chopra

**Affiliations:** ^1^Department of Biology, Center for Bionanotechnology and Environmental Research, Texas Southern UniversityHouston, TX, USA; ^2^Department of Microbiology and Immunology, University of Texas Medical BranchGalveston, TX, USA; ^3^Sealy Center for Vaccine Development, University of Texas Medical BranchGalveston, TX, USA; ^4^Institute of Human Infections and Immunity, University of Texas Medical BranchGalveston, TX, USA; ^5^Galveston National Laboratory, University of Texas Medical BranchGalveston, TX, USA

**Keywords:** simulated microgravity, *Yersinia pestis*, type three secretion system, high aspect ratio vessel, low shear forces

## Abstract

Manned space exploration has created a need to evaluate the effects of space-like stress (SLS) on pathogenic and opportunistic microbes. Interestingly, several Gram-negative enteric pathogens, e.g., *Salmonella enterica* serovar Typhimurium, have revealed a transient hyper-virulent phenotype following simulated microgravity (SMG) or actual space flight exposures. We have explored the virulence potential of *Yersinia pestis* KIM/D27 (YP) following exposure to mechanical low shear forces associated with SMG. Our experimental results demonstrated that SMG-grown YP was decreased in its induced HeLa cell cytotoxicity, suggesting that SMG somehow compromises T3SS functions. This was confirmed by an actual reduced amount of effector protein production and secretion through the T3SS injectisome. Also, SMG-grown YP proliferated less than their NG-grown counterparts did during an 8-h macrophage infection. Presently, we are evaluating the influence of SMG on various KIM/D27 mutant strains to further understanding of our initial phenomenology described above. Taken together, characterizing YP grown under the low shear forces of SMG can provide new insights into its pathogenesis and potentially uncover new targets that could be exploited for the development of novel antimicrobials as well as potential live-attenuated vaccines.

## Introduction

**Figure 1:**
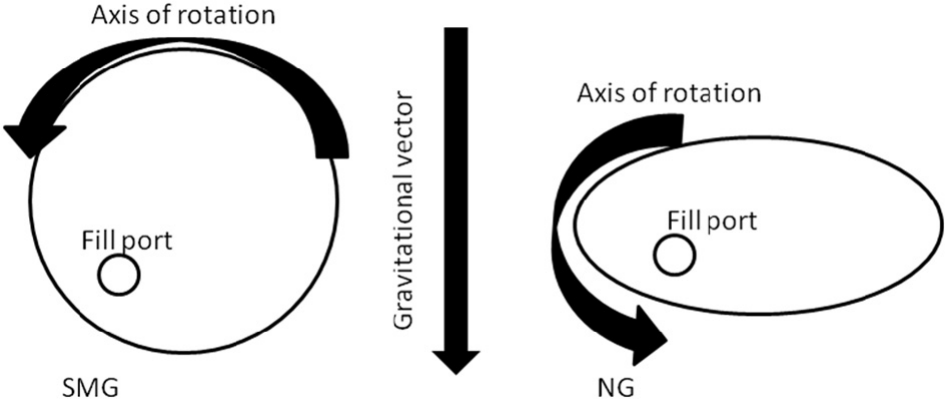
**High aspect ratio vessel induces SMG.** If the axis of rotation is perpendicular to the gravitational vector, SMG forces are achieved by which the gravitational vector force is randomized over the surface of the cells. If, however, the axis of rotation is parallel to the force of gravity, NG growth is achieved.

With our space flight programs, arose a shared concern of scientists and astronauts with the potential colonization of microbes, particularly bacteria, in our spacecrafts and eventually the International Space Station (ISS); however, of greater concerns are pathogens' communicability among astronauts in space (Kass, 1971; Rosenzweig et al., 2010). As a result, much emphasis has been placed on characterizing spaceflight-flown or simulated microgravity (SMG)-grown opportunistic pathogens that represent members of the human microbiota. Due to the low frequency of shuttle missions and their associated high costs, alternative ground-based methods for studying modeled microgravity had to be developed. To address this need, National Aeronautics and Space Administration (NASA) researchers developed a rotating wall vessel (RWV) or high aspect ratio vessel (HARV) which randomizes the gravitational vector over the surface of the cells being cultured, creating a SMG environment in which cells experience a state of constant “free-fall.” When the axis of HARV rotation is perpendicular to the force of gravity, then the aforementioned SMG condition can be achieved; however, if the HARV is repositioned such that the axis of rotation is in parallel with the force of gravity, then normal gravity (NG) conditions can be generated, often used as an experimental controlled condition (Figure 1). HARVs are commercially available and can be used to culture prokaryotes, adherent eukaryotic cells (with agarose beads), or suspension cells (Synthecon Inc. Houston, TX).

In addition to opportunistic pathogens such as *P. aeruginosa* (Crabbé et al., 2008, 2011), several bona-fide pathogenic bacteria, including *Salmonella* Typhymurium (Nickerson et al., 2000; Chopra et al., 2006; Wilson et al., 2007), entero-toxigenic *E. coli* (ETEC; Chopra et al., 2006), entero-pathogenic *E. coli* (EPEC; Chopra et al., 2006) and adherent-invasive *E. coli* (AIEC; Allen et al., 2008) have also been characterized following either space-flight or during SMG growth. The general trend observed has been a transient hyper-virulence and enhanced biofilm production, as seen in SMG-grown and spaceflight-flown *Pseudomonas* (Adams and McLean, 1999; McLean et al., 2001; Rosenzweig et al., 2010). With regards to ETEC, SMG enhanced its virulence potential through an up-regulation of the heat-labile (LT-1) enterotoxin leading to increased fluid secretory responses in the ligated ileal loops of mice (Chopra et al., 2006) whereas SMG-grown AIEC became hyper-adherent to a gastrointestinal epithelial cell line (Allen et al., 2008). Raising additional concern is the observation that space flight (and its various-associated conditions, e.g., microgravity and ionizing space radiation/solar flares) has been demonstrated to suppress immune system functions by promoting a T-helper cell 2 (Th2) bias. More specifically, T cells characterized following spaceflight demonstrated decreased production of both interleukin-2 (IL-2) and interferon gamma (IFN-γ) (Crucian et al., 2008).

Unlike the above mentioned closely related Gram-negative enteric pathogens, *Yersinia pestis* (YP) had not previously been characterized following either space flight or SMG conditions. YP is one of three Gram-negative pathogenic yersiniae, the latter also include *Y. pseudotuberculosis* (YPT) and *Y. enterocolitica* (YE), and they are members of the *Enterobacteriaciae* family, to which the aforementioned *S.* Typhymurium also belongs. Considering the fact that there is no currently available effective YP vaccine, as well as naturally occurring multiple-antibiotic-resistant strains (Rosenzweig et al., 2011a) novel chemothereputics are being developed as anti-plague counter-measures (Rosenzweig et al., 2011b). Therefore, learning all we can about YP pathogenesis, including how it responds to low shear force (in the form of SMG), could reveal novel chemotherapeutic targets and/or enhance the development of a novel, live-attenuated vaccine candidate strain.

All three pathogenic yersiniae harbor a well characterized ~70-kb virulence plasmid (pCD1 or pYVe) that encodes the type three secretion system (T3SS) injectisome as well as its effector protein substrates (*Yersinia* outer proteins-Yops). The T3SS is essential for YP virulence (Straley and Brubaker, 1982; Perry et al., 1998; Cornelis and Van Gijsegem, 2000; Deng et al., 2002; Viboud and Bliska, 2005; Dean, 2011). The T3SS directly translocates/injects effector Yops into targeted host cells orchestrating numerous anti-host effects, ranging from cell signaling disruption and paralysis to apoptosis.

Although unlikely to be found onboard a spacecraft or in the ISS, YP is bona-fide Gram-negative pathogen and evolved from YPT, it should also be characterized following growth under LSF (Rosenzweig et al., 2010). This chapter will discuss the YP response to space-like stress (SLS), namely SMG/LSF as it relates to its virulence potential as well as cross-reference the SMG responses of related pathogens providing an overview in the field.

### *S. enterica* space flight and SMG responses

The human pathogen best characterized in response to space-flight or SMG has been *S. enterica* (Nickerson et al., 2004; Rosenzweig et al., 2010), as evidenced by published studies of several groups and two space-flight experiments (Wilson et al., 2007, 2008). Both flight and ground-based SMG studies revealed differential gene expression (Wilson et al., 2002a,b, 2007; Chopra et al., 2006) and down-regulation of both T3SS-associated genes found in *Salmonella* pathognenicity islands 1 and 2 (Wilson et al., 2002a,b). These findings were at odds with the transient hyper-virulent phenotype observed in either space-flown or SMG-grown *S. enterica* resulting in both increased invasion of cultured cell lines as well as decreased LD_50_ relative to NG-grown *Salmonella* (Nickerson et al., 2000; Chopra et al., 2006; Wilson et al., 2007). Taken together, these findings suggested that SMG, validated by bona-fide space-flight virulence studies, was enhancing the *Salmonella* virulence potential in a T3SS-independent manner by an alternative, unknown mechanism. One potential explanation could be increased expression of the *virK* gene in SMG-grown *S*. Typhimurium which could promote increased bacterial virulence, as VirK contributes to remodeling of the bacterial outer membrane in response to the host environment (Chopra et al., 2006).

### Hfq as a global responder to SMG

While trying to identify space/SMG-responsive genes to uncover mechanistic clues as to how bacteria alter their virulence potential in response to SMG, one gene product emerged as a putative SMG-regulator on several occasions. Following space flight, many of the differentially expressed genes in *S*. Typhimurium were found to be part of the Hfq regulon (Wilson et al., 2007). Hfq is an RNA binding protein which has been implicated as a virulence-associated gene product in numerous notable pathogens including the *Yersinia* spp., *Salmonella* spp., *Vibrio* spp., etc. (Chao and Vogel, 2010). Taking into consideration that the Hfq regulon is differentially expressed in both *P. aeruginosa* (Crabbé et al., 2011) and *S.* Typhimurium (Wilson et al., 2007) following space-flight strongly suggests that Hfq is a space-responsive gene necessary for rapid bacterial re-programming in the aforementioned environment. Interestingly, when *Staphylococcus aureus* was grown under SMG conditions, it also exhibited down-regulated expression of *hfq* (Castro et al., 2011) in a manner consistent with what was formerly observed in *P. aeruginosa* and *S*. Typhymirium (Wilson et al., 2007; Crabbé et al., 2011). However, unlike the two aforementioned bacterial species, the *S. aureus* response to SMG resulted in decreased virulence potential (Castro et al., 2011).

Since SMG enhanced the virulence potential of Gram-negative pathogens through seemingly T3SS-independent mechanism(s) and via enhanced biofilm formation (Rosenzweig et al., 2010), we sought to evaluate the effect of SMG on a previously uncharacterized Gram-negative pathogen, YP KIM/D27.

### The YP virulence potential following SMG growth

Following SMG-growth, our first question was whether YP experienced any alteration/enhancement in its virulence potential. To measure this directly, we employed a previously published cell culture infection method in which bacterial proliferation is measured vis-a-vis cultured mouse macrophage-like cells, RAW 264.7 (Rosenzweig et al., 2005, 2007). Interestingly, SMG-grown YP demonstrated no significant difference in RAW cell proliferation relative to NG-grown YP counterpart (Lawal et al., 2010). In fact, fold changes in SMG-grown YP over the course of the 8-h infection period were actually lower than NG-grown YP. This strongly suggested that SMG might not influence YP in a manner similar to other Gram-negative pathogens tested, specifically *S*. Typhimurium which exhibited transient hyper-virulence phenotype in both cell culture and murine models of infection (Nickerson et al., 2000; Chopra et al., 2006; Wilson et al., 2007). Unexpectedly, it seemed to us that our cell culture infection data was suggesting the SMG was potentially reducing the virulence potential of YP rather than enhancing it.

### Reduction of SMG-grown YP virulence potential was a result of altered T3SS function

To determine whether YP indeed did not experience enhanced virulence potential following SMG-growth, we again employed a previously published method of cell culture infection that measures T3SS mediated host-cell cytotoxicty (Rosenzweig et al., 2005, 2007). HeLa cell rounding was compared following infection with SMG-grown YP, NG-grown YP, or an isogenic Δ *yopB* deletion mutant that is incapable of delivering/injecting effector Yops into the targeted HeLa cells. As a result, the Δ *yopB* deletion mutant served as a negative control. Interestingly, and in agreement with the above mentioned difficulty in proliferation vis-a-vis macrophages, SMG-grown YP appeared less apt in inducing HeLa cell rounding relative to its NG-grown counterpart. In fact, the reduced rounding mirrored the low levels of rounding observed when HeLa cells were infected with the negative control (Lawal et al., 2010). Being an indirect measure of T3SS function, we interpreted the rounding assay results to mean that SMG somehow impaired T3SS function. Further, this impairment could account for reduced proliferation when encountering host cells as well as a reduction in induced host cell cytotoxicty.

### Direct measure of T3SS function

Although the cytotoxicty assay described above strongly suggested T3SS impairment, the T3SS function itself had to be specifically measured to definitively make any statements. We then employed a previously published method that enabled us to directly measure the amount of Yops that were being produced and secreted. Interestingly, when we compared supernatant fractions of NG and SMG-grown YP, we observed a dramatic reduction in secreted YopM, one of the six Yop effector proteins. This demonstrated a deficiency in the secretion function of the injectisome needle itself in SMG-grown YP. To evaluate whether YopM levels were reduced in SMG-grown YP, we evaluated bacterial pellets and determined that the decreased secretion of YopM did not represent impairment of injectisome function but rather likely its reduced production within the cell (Lawal et al., 2010). By which mechanism LSF associated with SMG could be repressing T3SS-related gene expression remains unknown. However, it is of interest to note that following SMG growth, both *S*. Typhimurium and YP T3SS expression appears to be reduced. Despite a similar reduction in T3SS expression in both *S*. Typhimurium and YP following SMG growth, the former experienced transient hyper-virulence while the latter experienced hypo-virulence (Wilson et al., 2002a; Lawal et al., 2010).

## Conclusion

To date, there have only been two published exceptions to the prevailing trend of SMG-induced hyper-virulence in bacterial pathogens: YP (Lawal et al., 2010) and *S. aureus* (Castro et al., 2011). More specifically, SMG-grown YP was reduced in its proliferative capacity vis-a-vis cultured macrophages and suffered compromised T3SS function as evidenced by diminished induction of HeLa cell rounding. When the T3SS itself was specifically interrogated following SMG growth, reduced secretion of effector protein YopM was observed. YopM reduced secretion was later found to be a direct result of reduced YopM production within the cell. Ultimately, it is likely the reduced expression of T3SS-associated genes that is accounting for YP's reduced virulence potential following growth in SMG conditions; however, reduced expression of other virulence-associated genes could also have contributed to the reduction in virulence (Lawal et al., 2010). What remains unclear is why the closely related *S*. Typhimurium that also exhibited reduced expression of T3SS gene expression following growth in SMG (Wilson et al., 2002a,b) experienced hyper-virulence phenotype while YP experienced the seemingly opposite effect. Perhaps the explanation lies, in part, due to the fact the *S*. Typhimurium is a faculatative intracellular pathogen, while YP, although able to survive intra-cellularly, prefers to remain extracellular during infection (Lawal et al., 2010).

Taken together, space microbiology is a field in its infancy, and our expanding knowledge of SMG-influence on bacterial phenotypes will not only influence prolonged manned space explorations but also will broaden our understanding of bacterial pathogenesis on Earth.

Such an understanding could influence clinical decisions regarding when to administer chemotherapeutics in infected patients. Currently, too few pathogens have been evaluated following SMG-growth, and that list should be expanded for reasons discussed above. In fact, studies should be expanded to include additional Gram-positive pathogens and even fungal pathogens. A broader survey of both prokaryotic and eukaryotic microbial pathogens would catalog information that would be useful not only for astronauts with limited medications and no access to health care facilities in flight but also to the infirmed on the ground.

### Conflict of interest statement

The authors declare that the research was conducted in the absence of any commercial or financial relationships that could be construed as a potential conflict of interest.
